# Image processing and artificial neural network based determination of surface mean texture depth on lab-controlled chip seal pavement samples

**DOI:** 10.1038/s41598-024-78346-x

**Published:** 2024-11-13

**Authors:** İslam GÖKALP, Volkan Emre UZ, Mücahid BARSTUĞAN, Mehmet Can BALCI

**Affiliations:** 1 Faculty of Engineering and Architecture, Civil Engineering Department, Batman University, Batman, Turkey; 2 İzmir Institute of Technology, Faculty of Engineering, Civil Engineering Department, İzmir, Turkey; 3 Faculty of Engineering and Natural Sciences, Electrical and Electronics Engineering Department, Konya Technical University, Konya, Turkey

**Keywords:** Surface texture, Sand Patch Test, Hydrotimer, Image Processing, Surface void ratio, Artificial neural network, Civil engineering, Structural materials, Imaging and sensing

## Abstract

Because surface texture is nearly the sole indicator of pavement functional properties and highly correlated with critical operational characteristics of roadways like traffic noise and safety, the change in pavement surface texture because of traffic loadings and environment has to be evaluated routinely. There are numerous direct or indirect evaluation techniques in the market. However, most of these methods have some limitations like requiring lane closure or being expensive. In this study, a 2D image processing method was established to estimate the surface mean texture depth (MTD) of chip sealed pavements. We produced chip sealed pavement samples in the laboratory with different aggregate type, shape, and size ranging between 2 and 19 mm to cover wide range of live conditions. Two well-known conventional test methods, Sand Patch (SP) and Hydrotimer (HT), were used to determine MTDs of chip seal samples. Subsequently numerous photos were taken on surface of the samples with a camera for 2-D image processing that was done based on surface void ratio (SVR) approach. With the image processing, SVR of all samples were determined. At the point of whether there is a relationship or not, correlation analysis was made between the MTDs obtained with SP and HT and the data obtained by SVR approach with the artificial neural network method. The results show that the proposed SVR approach construed on 2D image processing method can be a reliable alternative to evaluate the surface texture of pavements.

## Introduction

Pavements are one of the most important civil infrastructures at the point of providing a sustainable dynamic life to the society^1^. They must provide a comfortable travel and/or driving experience as well as being safe for vehicles, pedestrians, and other user. Ensuring safety and comfort in traffic is a phenomenon that depends on the surface features of road pavements. Mainly, pavement surface characteristic is surface texture that domain numerous performances of pavement including skid resistance, drainage, noise level, fuel consumption, tire wear, emissions, and pollution impact. Therefore, it is a performance indicator as well as being an important feature that should be monitored regularly and intervened immediately when it reach the thresholds identified in specification for providing safety in road in service^2–7^.

Surface texture types are categorized as micro, macro, mega-texture and evenness that are defined as the deviation of a surface from a true planar surface depending on the magnitude of wavelengths varying between 0 and 500 mm and the ones larger than 500 mm^8,9^. Each one of these components has certain different influences for operational characteristics of a pavement. Micro-texture (with a wavelength up to 0.5 mm) is associated with the aggregate morphology, which dominate the microstructure of aggregate and then effect the dry-weather skid resistance of the road and tire wear, while macro-texture (with a wavelength from 0.5 mm to 50 mm) is linked with aggregate gradation and orienting of them on pavement surface. This texture influence drainage, noise level, and wet-weather skid resistance of the road. On the other hand, mega-texture (with a wavelength from 50 mm to 500 mm) can be linked to rolling resistance, in-vehicle noise, and vehicle wear. The final one that is evenness (with a wavelength longer than the upper limit of mega-texture) mainly influence ride quality^10–14^. As can be indicated that surface texture is vitally important for pavement performance and thus, an important object should be followed regularly and intervened while necessary.

There exist numerous methods proposed worldwide from the past to the present to estimate surface texture. These methods are commonly categorized as direct or indirect techniques depending on their implementation principle for assessment of the surface texture. The direct techniques can be called as conventional ones, which applied on the surface by an operator such as Sand patch (SP) and Hydrotimer (HT). The indirect ones are more technological and applied with the help of an operator but with machines with autonomous systems such as circular track meter, vehicle mounted profilometer^11,15,16^There exits certain limitations of the most of the current techniques to quantify texture although there are some advantages including simple principle and low cost. These might be as following: (1) they can only be used for fixed point measurement, (2) they are not only affected by human factors, but also by road surface factors and climatic conditions such as moisture and water seepage, and (3) they have low repeatability and efficiency^17–19^.

For the mentioned reason, researchers have developed some innovative methods to estimate surface texture using advantage of technological development. Today’s technological development allows researchers to use numerous applications in pavement monitoring and analysis including intrusive sensing, image processing techniques, and machine learning methods^20–26^. Image processing techniques and machine learning-based assessment were taken into consideration throughout the scope of the current study. To make a clear sense about techniques in the literature, a brief review was done and in this respect some studies were summarized at the following.

Georgopoulos, et al^27^. developed an algorithm to evaluate roadway surface cracking that is able to approximate the experts’ judgment using digital pavement images. The method includes digital image processing techniques to provide suitable digital imagery as input to specialized software that determines objectively and fully automatically the type, the extent, and the severity of surface cracking for flexible road pavements. The results showed that the proposed method presented substantial agreement while compared with systematic visual ratings of existing pavement cracking carried out according to the internationally accepted requirements for airfield and road pavement of the Federal Aviation Administration.

Mataei, et al^28^. designed an innovative device to simulate the saturation condition of the pavement surfaces and acquire photos from them during drainage process following saturation. The aim was to investigate pavement surface drainage by an automated image acquisition and processing system. Throughout this study, an image processing method was implemented to produce proper indices for drainage quality assessment. The preprocessing and enhancement of images was performed with Shearlet transform. The rate of surface drainage progress was examined by three indices extracted from the images. Finally, pavements were classified into three categories according to the indices extracted for their surface drainage. Evaluating the performance of the proposed system with reference images using the confusion matrix presented with 86% accuracy.

Chen, et al^17^. proposed an automatic close-range photogrammetry system (ACRP) that constructed based on the three cameras close-range photogrammetry to obtain real time asphalt pavement texture information and accurately monitor to estimate the antiskid performance of the road pavement. Authors implied that the ACRP system provides real-time and effective road surface anti-skid information for subsequent safety braking of autonomous vehicle and improves the efficiency and accuracy of traditional close-range photogrammetry.

Puzzo, et al^8^. developed an innovative procedure based on volumetric calculation to calculate the digital Mean Texture Depth (MTD) starting from the Digital Surface Model (DSM) generated by the photos considering 20 different pavement surfaces with a total of 100 DSMs. The results indicated that the proposed MTD calculated with an image-based procedure can be used instead of the MTD measured with the sand patch method, since the two indices assume very similar values, where the coefficient of determination is 0.99 with 0.061standard error.

Liang, et al^29^. proposed a novel pavement MTD evaluation strategy in the light of 3D pavement data filtered by a new filtering approach. In this regard, the authors first obtained 3-D pavement surface point cloud data based on a self-developed instrument, and then proposed a new 3-D data filtering method. Subsequently, they proposed a reference surface integral method to evaluate the pavement MTD. Conclusively, the novel proposed hybrid filter method has been showed better filtering performance without human intervention, and the proposed method for evaluating pavement MTD is considered effective with the error of 3.28%.

Apart from what has been summarized above, numerous researchers have studied this topic. Some examples are given in the following. Miao, et al^30^. examine the degradation of pavement surface MTD by 3D image processing techniques and entropy theory, Ghaderi and Abedini^31^used digital image processing technique to evaluate the airport runway flexible pavement MTD, Goodman^32^quantify the pavement textural and frictional characteristics by digital image analysis. In light of the studies presented and the ones not included in the current paper have proved that image-based techniques are suitable for the characterization of the pavement surface MTD and such methods have to provide more accurate results than traditional techniques. Roy, et al^33^. proposed an image processing-based index to quantify the macro texture of pavement. They as a results, established relationships between the proposed face surface macro texture index (SMI) and MTD/MPD. Such relationships are trustworthy and can be used to forecast MTD, a widely used measurement of pavement surface construction quality, and MPD, an indicator of skid resistance at the network level.

## Materials and methods

### Materials

**Fig. 1:**
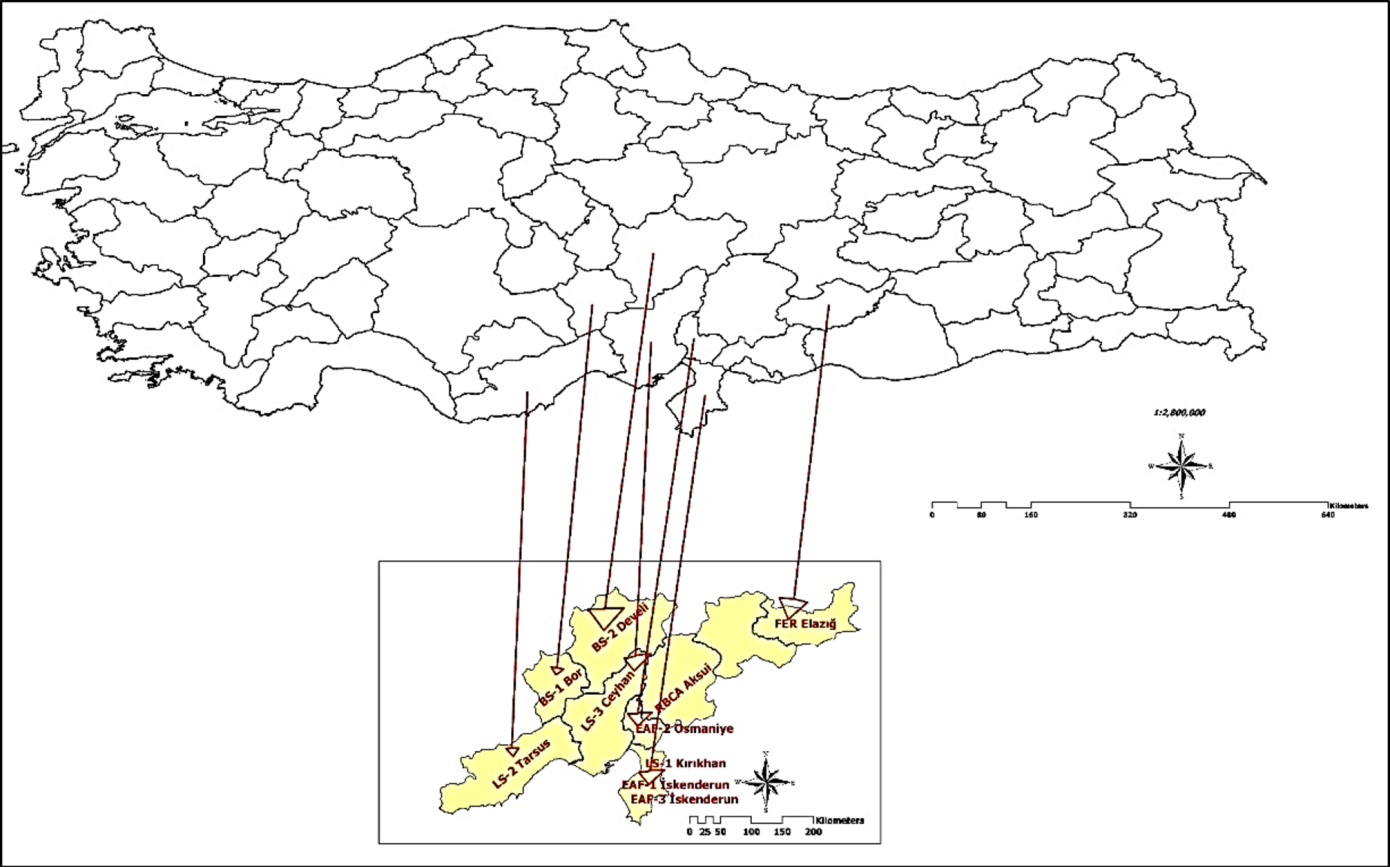
The map of region where aggregate supplied.

Different types of aggregates in origin including natural and industrial by-products were used within different gradations between 2.0 and 19.0 mm. The aggregate types used in the scope of this study were river basing crushed aggregate, limestone, basalt, ferrochrome slag, and electric arc furnace steel slag in order to have a wide range of pavement surface textures with different color that stimulate the real pavement surface under laboratory condition. The aggregates were supplied from different region of the country as pointed in the Fig. 1. The location map was generated with ArcGIS 10.2 (ESRI) software^34^ for the region of aggregate resources and with the manual of the program the map was created.

The physical and mechanical properties of aggregates were determined according to the aggregate test standards required for chip seal pavement in Turkish Highway Technical Specifications (HTS). The test results that were obtained from at least two repetitions and HTS limits are presented in Table 1.

**Table 1:** Properties of aggregates.

References		^35^	^36^	^37^	^38^	^39^		^40^			^41^		
Sample	Region	A (%)	B (%)	C (%)	D (PSV)	E (g/cm^3^)	F (%)	G (%)	G* (%)		H (%)	H* (%)	
LS-1	Kırıkhan	10.6	24.4	2.4	41.2	2.67	0.44	4	3		80–85	85–90	
LS-2	Tarsus	21.3	16.2	3.0	43.2	2.69	0.24	3	1		80–85	95–100	
LS-3	Ceyhan	11.7	24.4	8.1	41.6	2.69	0.28	4	1		85–90	70–95	
BS-1	Bor	10.4	12.0	6.9	61.0	2.61	2.00	2	1		60–65	85–90	
BS-2	Develi	9.4	25.9	9.4	52.4	2.67	1.44	2	1		75–80	95–100	
RBCA	Aksu	11.3	17.5	6.2	57.9	2.73	0.90	3	2		60–65	95–100	
EAF-1	İskenderun	9.5	22.9	2.3	76.1	3.40	1.79	4	1		50–55	80–85	
EAF-2	Osmaniye	8.8	25.3	8.3	59.0	3.39	2.46	4	1		45–50	80–85	
EAF-3	İskenderun	12.3	29.7	3.7	54.1	3.31	2.92	2	1		55–60	80–85	
FER	Elazığ	7.6	16.5	6.1	61.7	2.93	1.10	2	1		85–90	90–95	
HTS [38]-		≤ 25	≤ 30	≤ 18	≥ 40	-	≤ 2.5	≤ 10			≤ 60		

A: Abrasion Resistance, B: Fragmentation Resistance, C: Weathering Resistance, D: Polishing Resistance, E: Dry Unit Weight, F: Water Absorption, G: Vialit Plate, H: Nicholson Stripping Test, * The test done with modified bitumen with 0.2% DOP (by weight of bitumen) LS: Limestone, BS: Basalt, RBCA: River Basin Crushed Aggregate, FER: Ferrochromium Slag, EAF: Electric Arc Furnace Steel Slag.

The General Directorate of Highways (GDH) of Turkey indicates bitumen types being used in construction of new roads or maintenance and rehabilitation of existing roads are identified as 70/100 penetration grade. The basic bitumen properties were determined with standard test methods specified in HTS of Turkey and the test results obtained from at least double repetition are given in Table 2.

**Table 2:** Properties of Bitumen.

Properties	Unit	Standard	Test results	Limitations
Penetration	dmm	EN 1426	76	70–100
Softening Point	^0^C	EN 1427	48	43–51
Mass change	%	EN 12607-1	0.6	Min 0.8
Retained penetration	%	EN 1426	49	Min 46
Retained Softening Point	^0^C	EN 1427	56	52–60
Flash Point	^0^C	EN ISO 2592	238	Min 230
Solubility	wt %	EN 12,592	99.2	Min 99

### Methods

#### Manufacturing process of chip seals

Chip seals produced with Single-sized aggregate on specifically designed stainless specimen plate with 0.025 m^2^ area. To determine the aggregate spread rates for producing chip seal samples, Eq. 1constructed on basis of the average least dimension that represents the expected chip seal thickness as aggregates lie on its flattest side^42^was used. However, flaky particles for all types of aggregate were eliminated based on the ASTM D 4791^43^ and EN-933-3^44^ standards to eliminate the difference in flaky particles during production of samples. Flakiness sieve sizes, average least dimension, and aggregate spread rates according to chip sizes used in the study are given in Table 3.

1 $$\:ASR=\frac{950}{ALD}$$

Where,

ASR refers to aggregate spread rates and.

ALD refers to average least dimension.

**Table 3:** Aggregates spreading rate and flakiness sieves.

Chip sizes (mm)	Flakiness sieves (mm)	ALD (mm)	ASR (ml)
2.0-4.75	-	3.4	100
4.0-6.3	-	5.15	150
6.3-8.0	4.0	7.2	210
8.0–10.0	5.0	9.0	260
10.0-12.5	6.3	11.3	330
12.5–16.0	8.0	14.3	420
16.0–19.0	10.0	17.5	510

Surface dressing design method^45^ was followed to determine bitumen rate. In this method, there are numerous parameters linked with factors including traffic, existing surface, climatic conditions, and shape of aggregates to determine the aggregate spreading rate. Table 4 showed the parameters and the factors value of surface dressing design.

**Table 4:** The parameters and factors of surface dressing design.

Traffic type	Vehicles (L/D)	F	Existing surface	F	Climatic condition	F	Type of chips	F
Very Light	0–50	3	Untreated or Prime Base	6	Wet and Cold	2	Round / Dusty	2
Light	50–250	1	Very Lean Bituminous	4	Wet and Hot	1	Cubical	**0**
Medium	250–500	0	Lean Bituminous	0	Temperate	0	Flaky	-2
Medium-Heavy	500–1000	-1	Average Bituminous	-1	Semi-Arid (Hot and Dry)	-1	Pre-Coated	-2
Heavy	1500–3000	-3	Very Rich Bituminous	-3	Arid ( Very Hot and Very Dry)	-2	-	-

Following the determination of ALD of the aggregate, the binder application rate can be determined by using Eq. 2.

2 $$\:R=\:0.0625+(F\times\:0.023)+(0.0375+(F\times\:0.0011\left)\right)\times\:ALD$$

where,

R is basic spreading rate of bitumen in unit of kg/m^2^ and.

F is weighting factors.

With respect to the factors presented in Table 4, the determining factors were medium for traffic, lean bituminous for existing surface, temperate for climate, and finally cubical for aggregates. Accordingly, total factor (F) is calculated as zero. Subsequently, bitumen application rates for each aggregate size and type are calculated based on the plate surface area and the results are presented in Table 5.

**Table 5:** The amount of bitumen used to produce chip seals.

Chip Sizes (mm)	The amount of bitumen (ml)	
	Calculated	Used
2.0-4.75	18.4	19.0
4.0-6.3	19.9	20.0
6.3-8.0	21.5	21.0
8.0–10.0	23.0	23.0
10.0-12.5	24.9	25.0
12.5–16.0	27.4	27.5
16.0–19.0	30.0	30.0

**Fig. 2:**
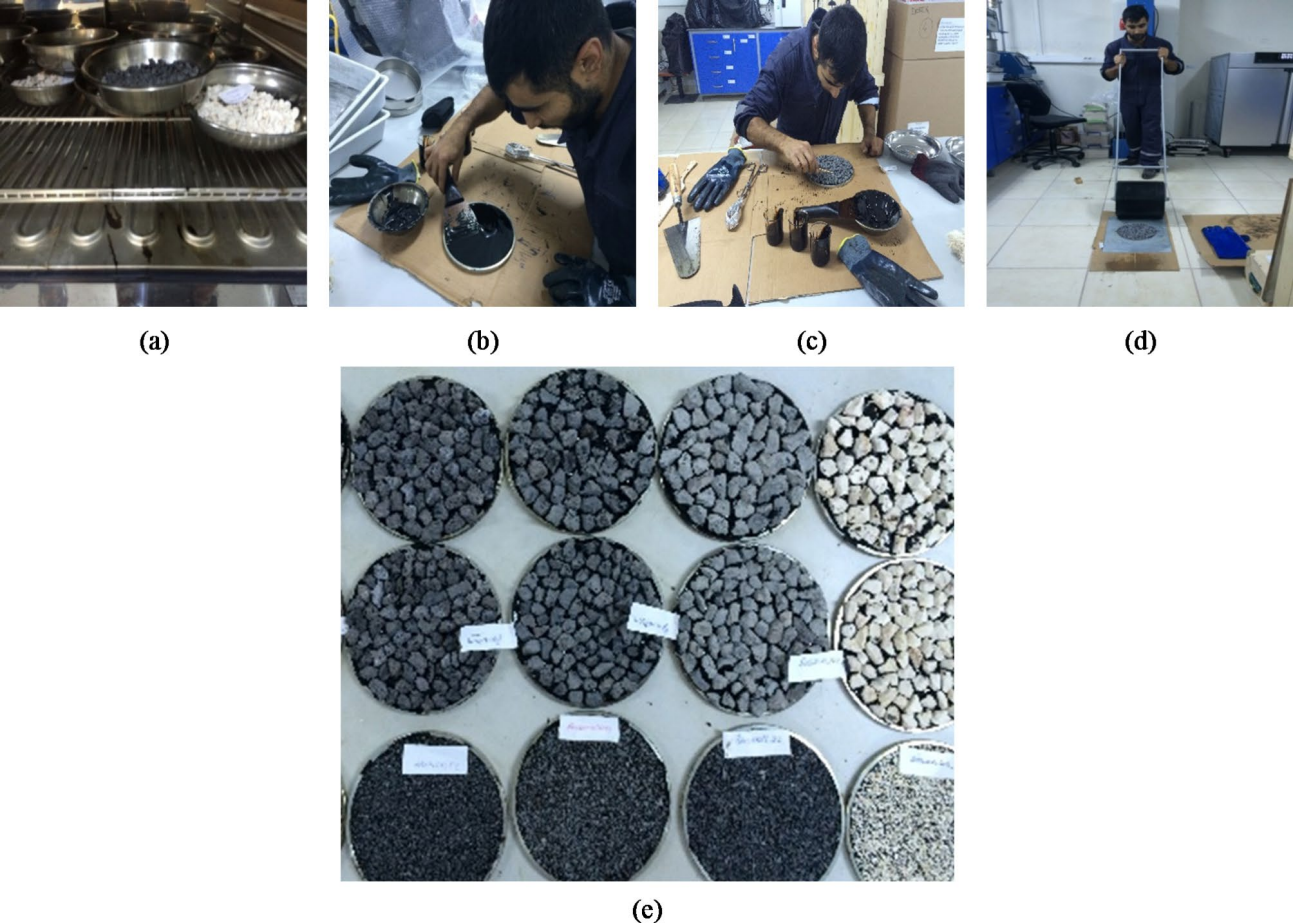
Manufacturing process for chip seal samples.

Following steps were followed in chip seal production. Some photos are taken to show during sample production are given in Fig. 2.

(1) Putting the standard sample plate and the measured aggregates into the ventilated oven at 100 ± 5 ^o^C for heating (Fig. 2-a).

(2) Heating the measured bitumen at 145 ± 5 ^o^C and the aggregates into the non-ventilated oven and pouring the hot bitumen on the hot plate, and lied with a spatula over the plate (Fig. 2-b),

(3) Spreading and orienting the aggregate on the plate with bitumen (Fig. 2 -c),

(4) Rolling with a rubber cylinder in both directions for three passes (Fig. 2 -d),

(5) Curing the prepared samples for 24 h at ambient temperature in the laboratory (Fig. 2-e).

#### Macro texture measurement

To determine the macro-texture of the chip seals, two well-known volumetric methods were used. The first one was Sand Patch and the second was Hydrotimer. These two methods are presented briefly in this part such as following.

##### Sand patch

It is used for determining the macro texture of pavement surface. The implementation of this test varies due to standards published by different agencies or institutions. In this study, ASTM-845E 965 − 12^46^ was used to operate the sand patch test. To implement the test 25 ml sand was used for chip seals having grain sizes lower than 6.3 mm, while 50 ml sand was used for chip seals having grain sizes bigger than 6.3 mm. To do the first, the surface is cleaned from the friable particles with a soft brush. Then the known volume of sand is poured onto a cleaned surface and spread out in a circular motion with the spreader. This spreading motion has been continued until the diameter of circle stabilizes, sand particles have completely filled the voids and the sand patch is leveled to the highest points on the surface. Afterward, the diameter of the circle was measured for four evenly spaced diameters. Finally, the average of four readings is determined and the mean texture depth is calculated by Eq. 3.

3 $$\:\text{M}\text{T}\text{D}=4\text{V}/{\uppi\:}{\text{D}}^{2}$$

where,

V refers to sand volume and.

D refers to average diameter.

##### Hydrotimer

**Fig. 3:**
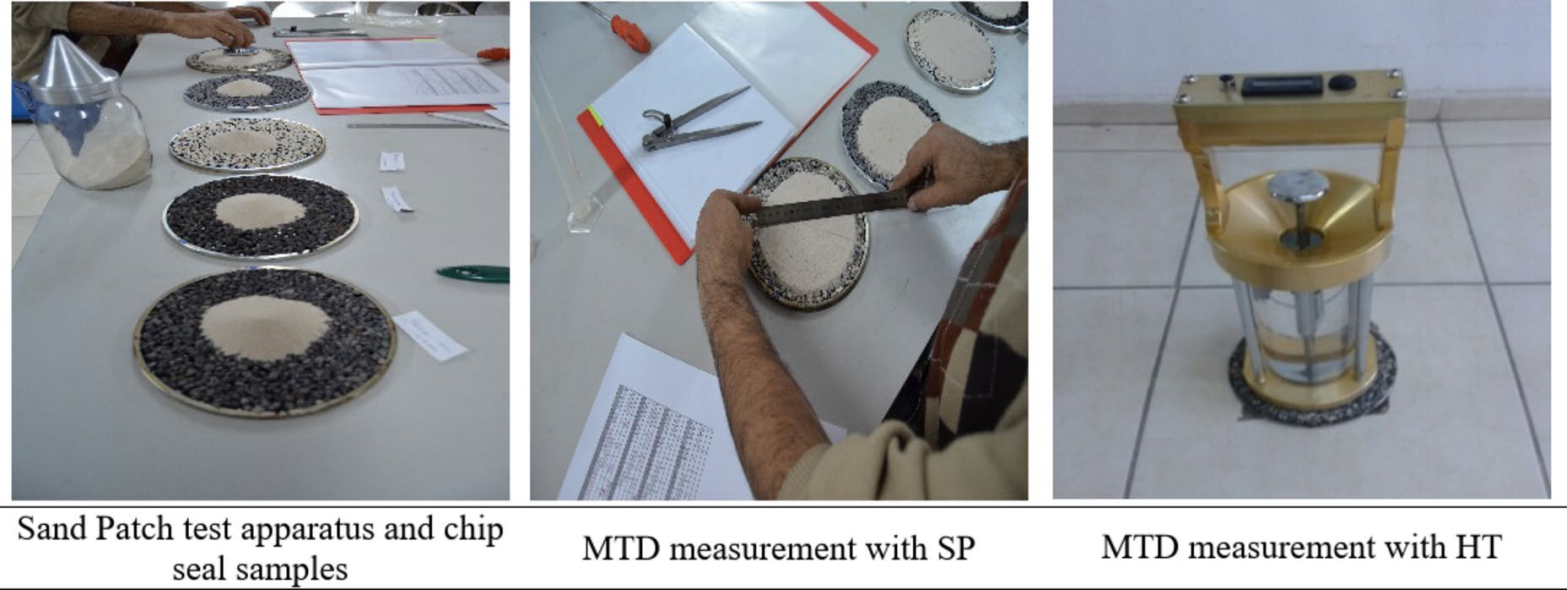
Test methods and devices.

It measures the time takes a known quantity of water to escape through voids in the samples was also used to determine the MTD according to ASTM E 2380-12^47^. The HT device is filled with water and placed on the sample in a plunger-sealed position, and the timer is reset to zero. To allow discharge of the water, the plunger is released. The timer is activated with the water level passing the upper float switch and stopped counting automatically when the water level passes the lower float switch. The flowing time of water between two switches is indicated on the timer and recorded by the operator. The test was repeated at least four times by relocating the device over the sample. The arithmetic average of the results is calculated with Eq. 4 for MTD. Numerous photos taken during application of macro texture test are given at the Fig. 3.

4 $$\:\text{M}\text{T}\text{D}=\left(\frac{3.114}{\text{O}\text{F}\text{T}}\right)+0.636$$

where,

OFT refers to average outflow time.

#### YOLOv4 algorithm

Deep learning models are powerful algorithms that have witnessed a recent increase in literature for detection, segmentation and recognition. The development of deep learning models, which were introduced by LeNet in 1998, ceased for a while due to the inadequacy of hardware, but resumed with the presentation of AlexNet, VGG, GoogleNet, ResNet to literature in 2012. Following the adoption of these methods for image classification, the need to quickly detect an object on an image, regardless of size difference, arose. With the proposal of the SSD, R-CNN, Fast R-CNN, and Faster R-CNN models, which are based on the Region-based CNN approach, algorithms with high accuracy and speed have been introduced to the field. These models divide the image into regions and search for the most suitable object within the appropriate regions after training. YOLO (You Look Only Once), another Region-based deep learning model, is a convolutional neural network (CNN) based deep learning algorithm that is fast enough to detect objects on the image in one go.

YOLO is constantly improving itself with different versions, and it is the YOLO4(v4) algorithm that was introduced in April 2020 that was used in the present study. The computational load is very high in classical CNN methods, while YOLOv4 has produced successful results in real-time applications compared to its competitors. The YOLO method uses regression to define object positioning with bounding boxes. The algorithm first divides the image into regions, and then searches for the desired object in that region. The confidence score is obtained by calculating the probability that the searched object will be in that region and presents the result as a percentage. However, over-estimations and the drawing of several bounding boxes around a single object can occur in studies. To prevent this, the non-maximum suppression technique is applied to the objects inside the bounding boxes. This technique excludes objects with a lower confidence score from the evaluation, and checks for the presence of a bounding box with a higher confidence score in the same region. In this study, the YOLOv4 algorithm was used to determine the aggregate size.

#### Image processing

**Fig. 4:**
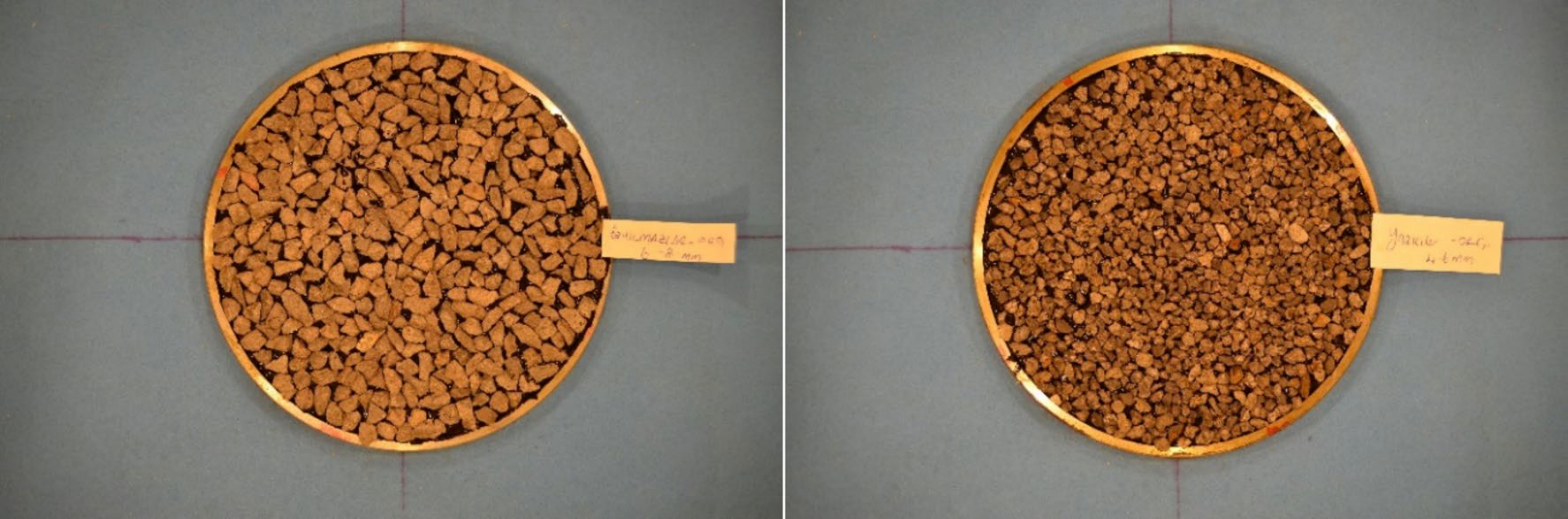
Chip seal image samples.

In the present study, photographs of chip seal samples taken using a high resolution camera (Nikon 5100) under a certain height and magnification in closed cubical box with standard lighting produced in a controlled manner in a laboratory. To illustrate the produced the chip seal sample, following images are given in Fig. 4.

Setting were processed using YOLOv4 and morphological image processing methods, as well as the Python High-level programming language and OpenCV library in a digital environment.

**Fig. 5:**
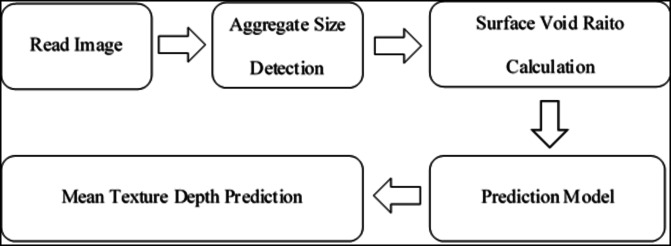
Aggregate detection and mean texture depth prediction phase.

In the image processing stage, the first image taken was resized to 1080 × 720, and the resized image was converted to HSV color space and subjected to threshold. Erosion and merger methods were then applied to the image, and aggregates were identified by finding the edges of the output image, while the surface void was determined by finding the voids on the surface. The process flow diagram obtained in the experimental results section is shown in Fig. 5.

**Fig. 6:**
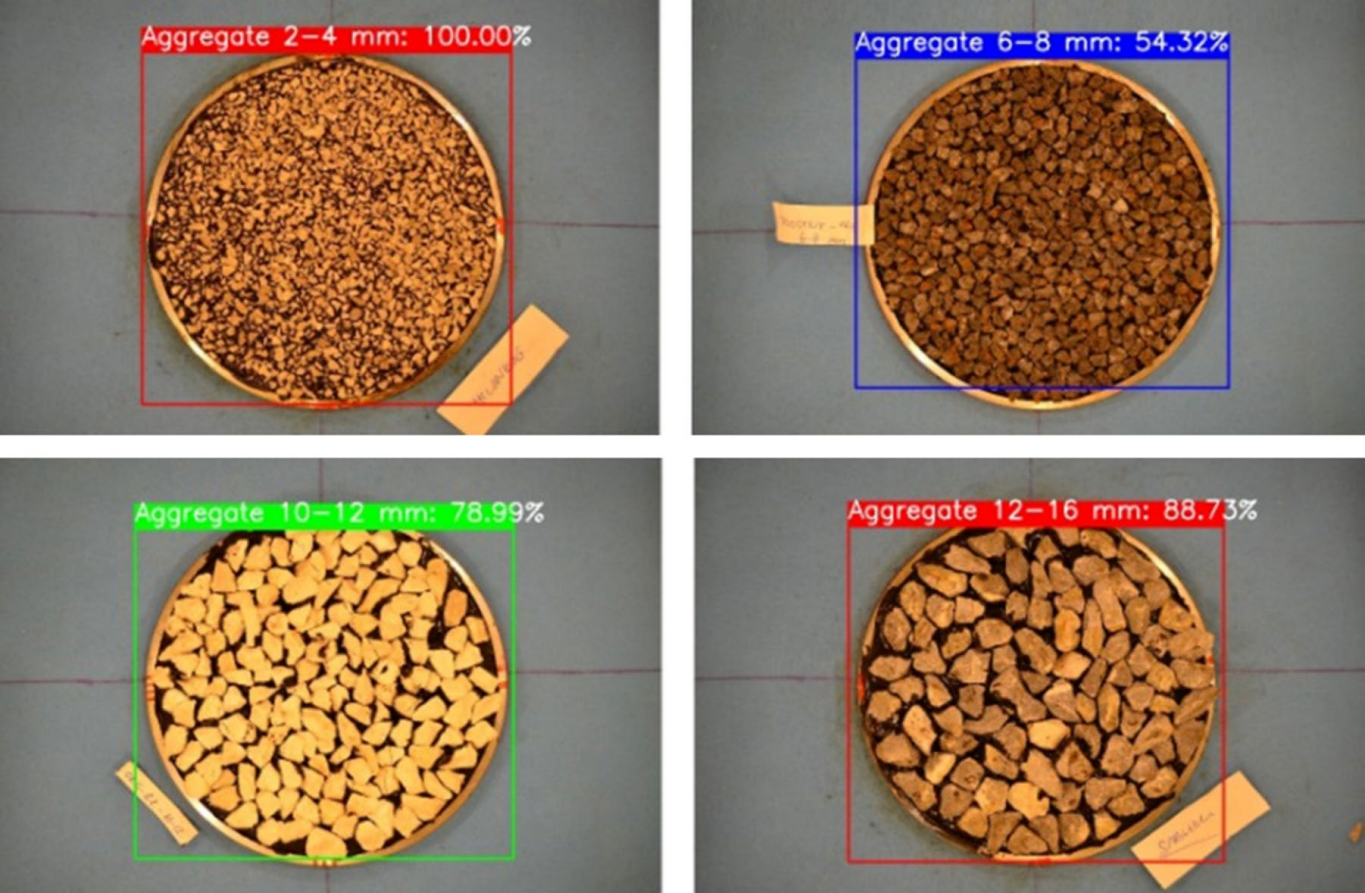
The aggregate identification results obtained using the YOLOv4 algorithm.

As can be seen in the figure, the aggregate size was first determined using the YOLOv4 algorithm. The following Fig. 6 presents some of the results in this respect.

**Fig. 7:**
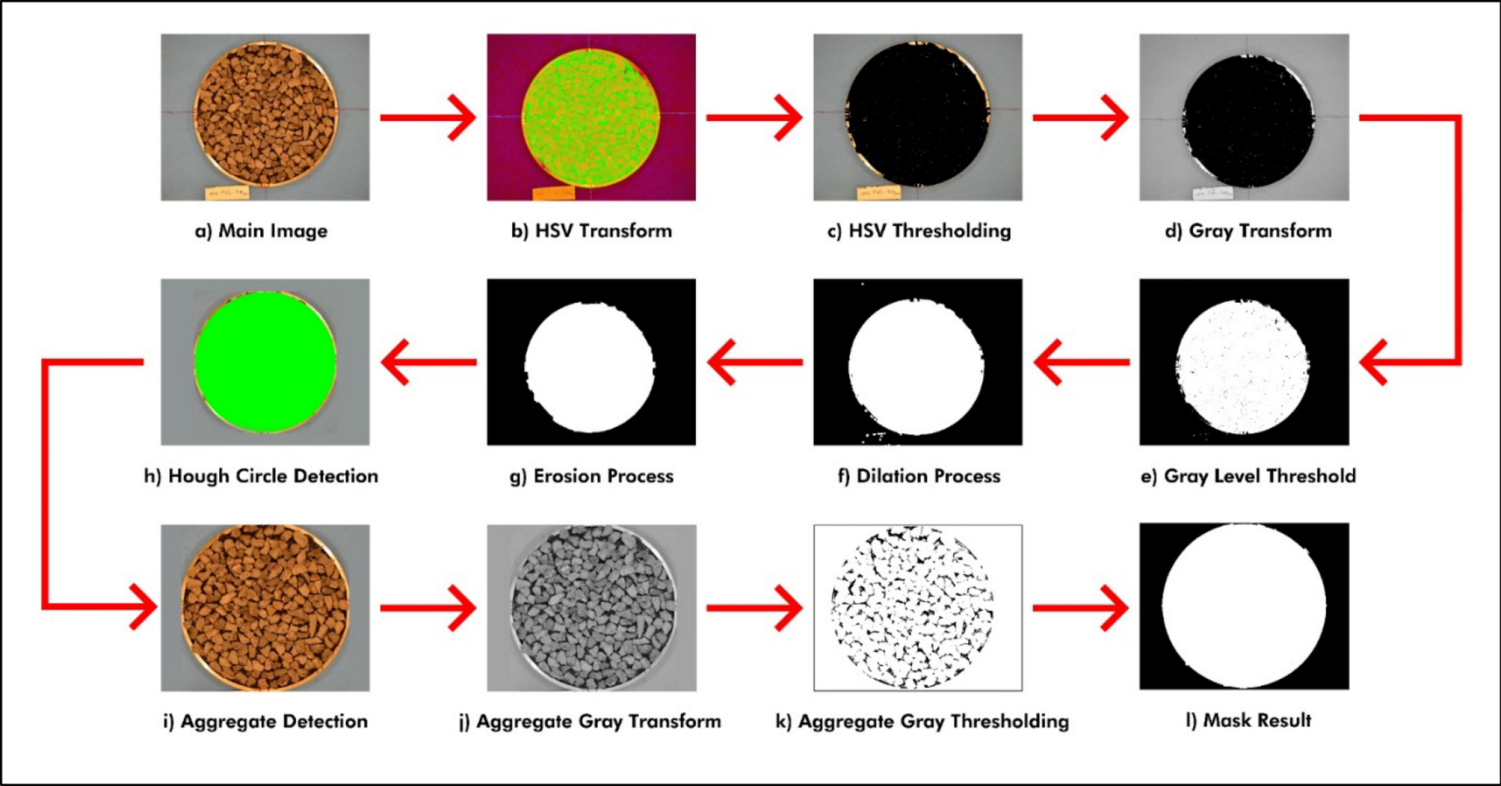
SVR approach from morphological image processing.

The aggregate identification results were recorded to estimate the aggregate size, after which the surface void ratio was found through morphological operations. This process is shown in Fig. 7.

**Fig. 8:**
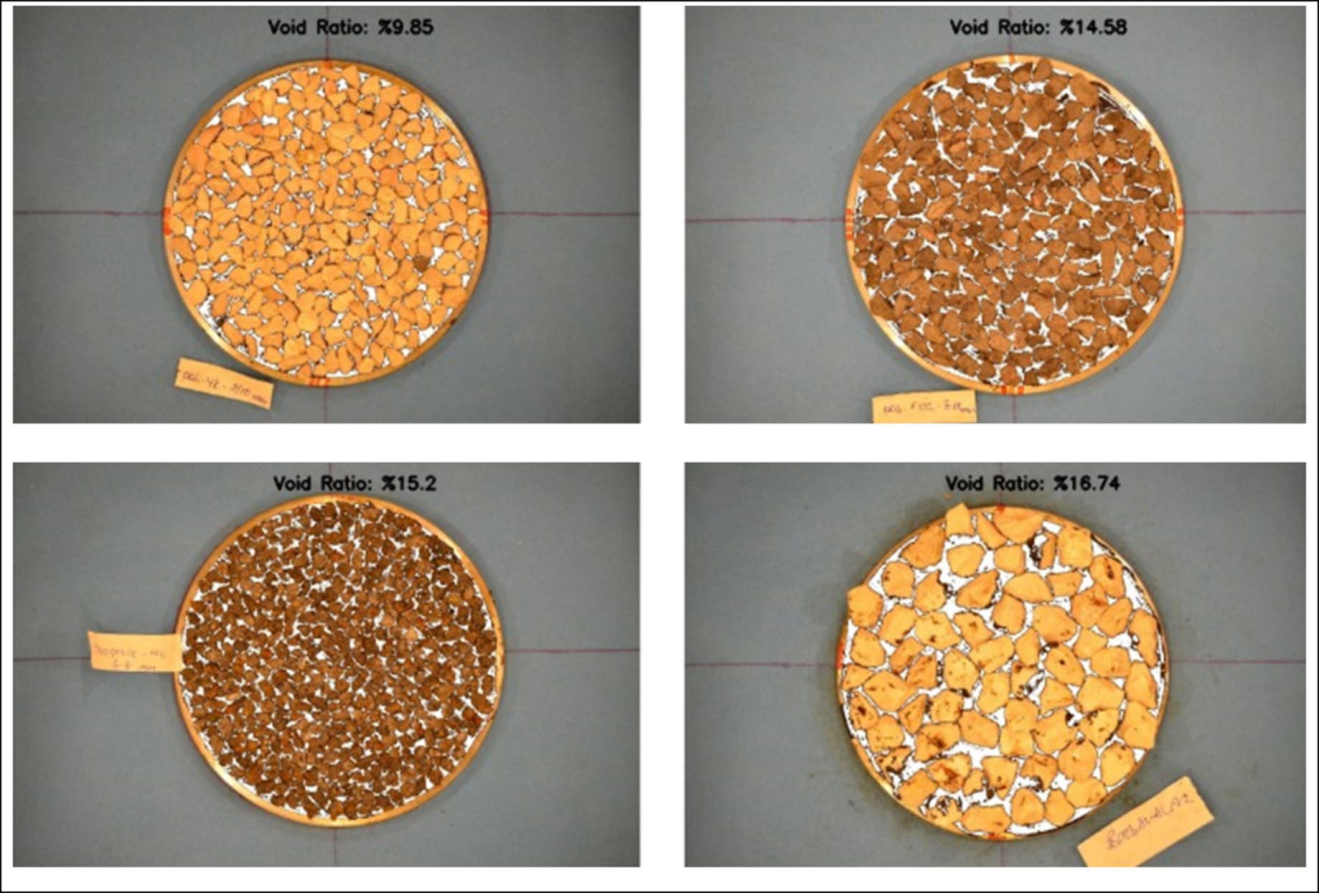
Examples of void ratio results.

The surface void ratio was found by comparing the image in Fig. 7-k with the aggregate mask (Fig. 7-l) that was prepared automatically during the algorithm. During the comparison, the number of black and white pixels within the circular area was found, and the void ratio was obtained by calculating the ratio of the number of black pixels to the total number of pixels. Some results are presented in Fig. 8.

#### Artificial neural networks (ANN)

**Fig. 9:**
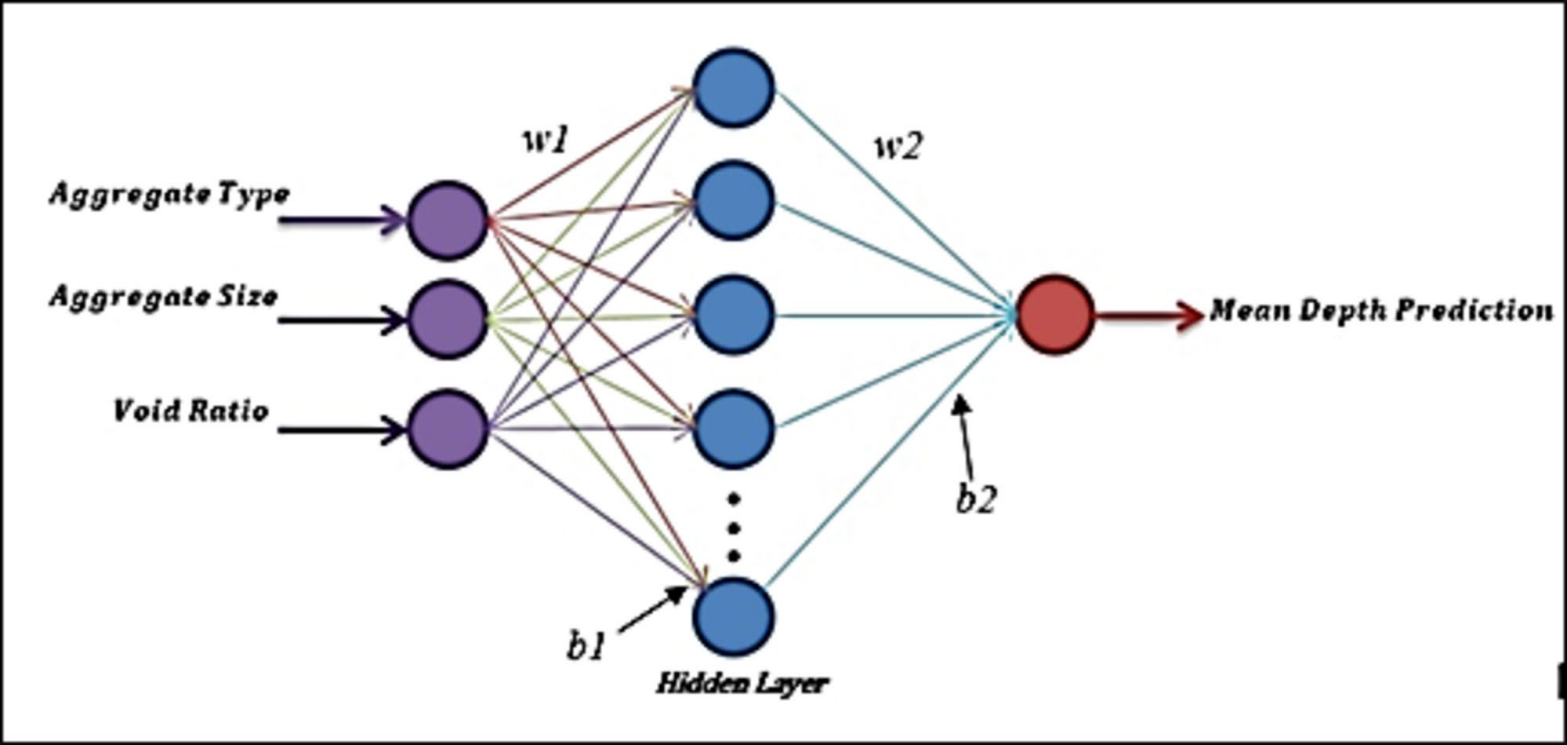
Example of ANN structure.

MTDs determined by SP and HT for chip seal samples and MTDs predicted by image processing were compared with using Linear Regression and Artificial Neural Networks (ANN) methods. ANN is a flexible mathematical modeling method that can learn system behaviors through the use of input and output datasets. It models the relationship (linear or nonlinear) between the inputs and target in any problem by generating acceptable solutions on examples that have not been seen or implemented before. ANN can be used to resolve problems in many different engineering branches given its ability to learn, its ease of application to different problems, its generalizability, the need for less information, etc. An ANN usually has an input layer, a hidden layer and an output layer, and there are as many neurons as the number of inputs in the input and output layers. An example of an ANN structure is shown in the Fig. 9.

In the ANN structure seen in Fig. 4. By training these values, the equation of the ANN model was obtained. The equation obtained as a result of ANN is presented in Eq. (5).

5 $$\text{MTD Prediction}=\text{b2}+\text{w2}* \text{tansig}(\text{b1}+ \text{w1}*\text{testdata})$$

where;

w1 and w2 represent the weights and.

b1 and b2 represent the bias values.

## Results and discussion

### MTD results

The MTD of the chip samples produced at laboratory conditions with different chip sizes ranging between 2.0 and 19.0 were determined with two known test methods, which were SP and HT. Measurement of the diameter of the circular sand that spread on the sample surface was taken from four different directions to determine the MTDs with SP. On the other hand, at least five repetitive OFM values were measured with the HT test method. The arithmetic mean of the values obtained for the reasonable test values that remained in standardized intervals and then MTDs for all chip seal samples on basis of the two test methods were calculated by the aforementioned equations given above. The arithmetic mean values of MTDs determined by SP and HT methods are presented in Tables 6 and 7, respectively.

**Table 6:** MTDs (mm) derived by SP.

Chip Size (mm)	LS -1	LS-2	LS-3	BS-1	BS-2	RBCA	EAF-1	EAF-2	EAF-3	FER
2.0-4.75	2.69	2.61	2.52	2.87	3.34	2.39	2.56	3.30	3.44	3.17
4.0-6.3	2.93	2.88	2.60	3.36	3.58	3.11	3.44	4.05	3.32	3.40
6.3-8.0	4.14	3.51	4.11	4.33	4.09	3.85	4.72	4.90	4.90	4.54
8.0–10.0	4.64	4.36	4.70	5.02	4.51	4.76	5.24	5.82	5.04	5.22
10.0-12.5	5.11	5.11	5.22	5.86	6.05	5.66	6.24	6.97	6.85	6.42
12.5–16.0	7.92	6.65	7.44	7.40	7.94	6.86	8.84	8.67	8.90	8.09
16.0–19.0	8.81	7.70	8.53	9.05	9.53	8.55	10.11	9.40	10.56	10.01

**Table 7:** MTDs (mm) derived by HT.

Chip Size (mm)	LS -1	LS-2	LS-3	BS-1	BS-2	RBCA	EAF-1	EAF-2	EAF-3	FER
2.0-4.75	1.96	1.70	1.72	1.88	2.16	1.59	1.69	2.31	2.39	2.28
4.0-6.3	2.01	2.22	1.97	2.86	2.42	2.29	2.71	2.62	2.22	2.98
6.3-8.0	3.14	2.47	3.27	3.00	3.26	3.17	3.31	3.14	3.47	3.26
8.0–10.0	3.86	3.42	3.40	3.47	4.02	3.85	3.58	3.92	4.11	4.26
10.0-12.5	4.11	4.76	4.64	4.14	5.20	4.36	5.60	4.91	5.35	4.56
12.5–16.0	6.82	5.50	6.38	5.60	6.49	4.70	6.32	6.67	7.07	5.10
**16.0–19.0**	**FM**	**FM**	**FM**	**FM**	**FM**	**FM**	**FM**	**FM**	**FM**	**FM**

FM: Fail in Measurement.

As can be seen from Tables 6 and 7 the MTD values increase as the chip size increases. The increasing rate is not uniform for all types of samples, but different from the origin of aggregate changes. The reason can be linked with the surface texture of aggregate, which are different for different aggregate. The other reason may be the ASR determined for each chip size that ranges from 100 to 510 ml as the chip size increases. MTDs determined with SP and HT are compatible with each other, but the values are different. Since the operation and calculation of MTD for both methods are different from each other. Due to the HT working principle, MTD for the chip seal samples produced 19.0–16.0 mm chip cannot be possible to determine. Because, the known volume of water discharge quite quickly, the time counter runs out before it works. In fact, this situation shows the applicability of HT in the chip seals produced with aggregates of what size, and respectively, it can be concluded that 12.5–16.0 mm size is the threshold for application of HT on chip seal type pavement.

It can be possible to determine MTDs of chip seal pavement with standard and well-known tests. However, too many measurements must be made at short intervals defined throughout specifications so that the mean texture depth of the pavement can be accurately determined in site application. If mean texture depth estimation can be made by processing a photograph taken from the pavement surface accurately, this will help to prevent the required labor and waste of time, and even the texture depth can be determined very quickly on a wide surface. With this in mind, the subject of this study was established, and studies were carried out to achieve this aim with the designed image processing method described above.

### Image processing analysis results

With the designed image processing system, it was aimed to predict the MTD with the SVR (%). Respectively, the SVR (%) for each chip seal sample determined by image processing is given in Table 8.

**Table 8:** SVR (%) of chip samples derived by image processing.

Chip Size (mm)	LS -1	LS-2	LS-3	BS-1	BS-2	RBCA	EAF-1	EAF-2	EAF-3	FER	SVR Interval (%)
2.0-4.75	8.66	15.20	19.83	17.30	17.30	18.72	25.41	26.91	25.51	22.15	8.66–26.91
4.0-6.3	17.19	17.11	15.16	15.49	15.08	8.57	19.28	23.21	16.83	19.90	8.57–19.90
6.3-8.0	13.23	16.97	9.85	13.38	15.00	12.04	16.48	18.05	22.12	17.15	9.85–22.12
8.0–10.0	15.70	12.85	12.47	14.58	11.63	12.65	13.33	17.91	13.43	14.58	11.63–17.91
10.0-12.5	15.05	11.87	13.63	10.89	12.09	13.06	11.29	19.31	12.65	13.97	10.89–19.31
12.5–16.0	15.00	21.47	20.39	15.92	15.92	15.57	15.07	19.97	20.78	19.11	15.00-21.47
16.0–19.0	19.02	21.84	18.56	19.63	19.63	18.02	16.74	17.24	21.19	23.08	16.74–23.08

The data given in Table 8 showed that the surface SVR of each sample are different from each other even though they were manufactured with the same chip sizes couple. The SVR values were not depending on the aggregate chip sizes but on the aggregate type. Because the SVR of the test samples with the same chip size couples’ changes with aggregate type even the ones with the same origin. The reason for it can be liked by the microstructure and the particle shape of aggregates. Since some have smooth surfaces and others have porous surfaces. This, effect the orientation of each particle, although the roller was passed over them to make the sample surface uniform.

### Artificial neural network (ANN) results

The SVR information obtained from the image processing result was added to the dataset developed for the training of the estimation model before moving on to the prediction model creation phase. Artificial neural network training involves many parameters, such as the number of iterations, training and activation functions, learning rate, the number of hidden nodes, and the number of hidden layers. The parameters used in the training of the network structure established in this study are presented in Table 9. Accordingly; training and test results for SP and HT are presented in Tables 10 and 11, respectively.

**Table 9:** Training phase parameters.

Parameter	Values
Number of Iterations	2000
Training Function	trainbr, trainbfg, trainscg
Activation Function	poslin, tansig
Number of Hidden Layers	1,2
Number of Hidden Nodes	10,20,30,40,50,60

**Table 10:** Training and test results for SP.

Structure	NoHL	NoHN	TF	Training *R*^2^ Value	Test *R*^2^ Value
Structure 1	1	10	tansig	0.9325	0.9101
Structure 2	1	20	tansig	0.9849	0.9331
Structure 3	2	30,30	poslin, poslin	0.9385	0.8765
Structure 4	1	40	poslin	0.9729	0.9719
Structure 5	2	50,50	tansig, tansig	0.9767	0.9444
**Structure 6**	**1**	**60**	**tansig**	**0.9764**	**0.9813**

The total number of chip seal sample is 60, 85% of which was randomly allocated to training and 15% to testing. During the training phase, different training and activation functions, and different numbers of hidden nodes (NoHN) and hidden layers (NoHL) were tested, and the results were compared. The best 6 results obtained in the study are presented. In the trials, the best training function was found to be “trainbr”; and the best activation function was found to be “poslin”. The present the prediction results obtained during the training and testing phase for HT and SP. As can be seen in Table 10, the best training result was calculated to be 0.9767, although the test result of the same structure revealed a test success of 0.9444.

**Table 11:** Training and test results for HT.

Structure	NoHL	NoHN	TF	Training *R*^2^ Value	Test *R*^2^ Value
Structure 1	1	10	tansig	0.9503	0.8860
**Structure 2**	**2**	**20**,** 20**	**tansig**,** tansig**	**0.8891**	**0.9189**
Structure 3	1	30	poslin	0.9617	0.9071
Structure 4	2	40,40	tansig, tansig	0.8752	0.9168
Structure 5	2	50, 50	tansig, tansig	0.9126	0.8674
Structure 6	1	60	tansig	0.8903	0.8658

**Fig. 10:**
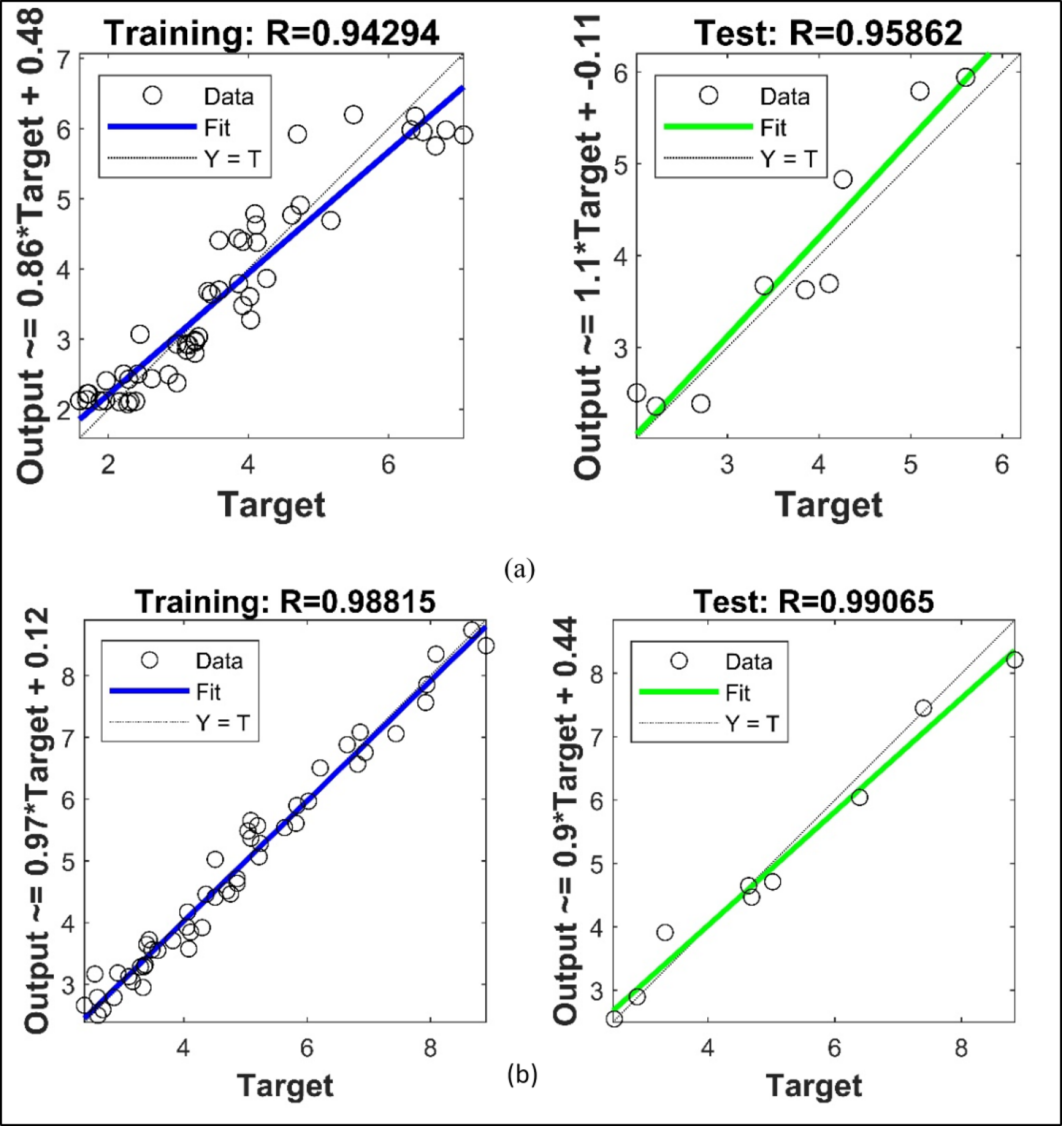
Results for SP Structure 6 (**a**), Results for HT Structure 2 (**b**).

When evaluating the test results of ANN structures, the test results should be higher than the training results. If the training result is higher than the test result, the result is an overfitting. It can thus be said that Structure 6, where test success is higher than training success, performs the best learning process. In Table 11, the best training result in HT data was found to be 0.9503 in Structure 1, although the test result was 0.8860. Overfitting occurred in this structure as well. The only structure in which no overfitting occurred was Structure 2, where the training result was 0.8891 and the test result was 0.9189. The data distribution and curves obtained for Structure 6 in SP data and for Structure 2 in HT data are shown in Fig. 10.

## Limitations

This samples evaluated in the current study was produced at laboratory with different aggregates have different color from light to black to capture the reality of color change on road pavements due to used aggregate and polishing under traffic service. The photos taken on the samples under specifically design closed box with daylight lighting. Although we want to create an exact replica of the situation on the road pavement in the laboratory, it cannot be said that this is 100% achieved.

## Summary and conclusion

In this study, it was aimed to estimate the MTD of chip seal pavement samples produced at laboratory with single grain sizes ranging from 2 to 19 mm with a standard method using a 2D image processing-based SVR approach. Throughout the scope of the study, ten types of aggregate in different origin with different color were used to produce standard chip seal samples. Properties of all aggregates samples were identified according to the material requirements given for chip seal pavement aggregate defined in HTS of Turkey. To determine the MTD of each samples, two well-known volumetric assessment based conventional test method, SP and HT tests were conducted ın each sample. Subsequently, photos of each sample were taken with the specified method for the aim of using in image processing to estimate MTD with the constructed algorithm on basis of the SVR approach. At the end of the study, the following conclusion can be highlighted.

- MTD values found with the conventional methods give different results although both were applied on the same samples,
- MTD values of each chip seal is depending on the chip size couples used in preparation of them.
- The chip seal samples prepared with 16.00–19.00 mm chip couples were not available for testing with HT due to quick drainage.
- It is found to be possible to predict the MTD of chip seal samples prepared within the scope of this study with 2D image processing.
- The 2D image processing-based SVR approach can be an alternative tool for the prediction of MTD instead of the two conventional MTD test methods.
- Using the SVR approach in MTD prediction provides certain benefits including saving cost and preventing loss of labor, and ensuring the roads serve at full capacity by not closure of the traffic lane.

With this study, it is thought that it will support the texture depth of different pavement types of the image taken with a single remote sensing method of the pavements from a different angle and the non-destructive test analysis can be established in future studies.

## Recommendations for future studies

In future studies, revealing this with photos to be taken on the road in daylight will make significant contributions to the SVR approach. Moreover, the scope of SVR approach application can be expanded with the evaluations to be made on the surface pavements to be produced with different aggregate gradation, on the roads built with bituminous hot mixture, instead of the controlled samples produced with single-size aggregates.

As briefly summarized above, the study was established and examined under laboratory conditions with controlled laboratory samples. However, to expand the results and the use of the SVR approach in this study under real traffic case, there will be worth to study on the samples taken at field. This may minimize the uncertainty created by the results obtained from controlled laboratory samples. Moreover, instead of analyzing single size chip seal, investigation on chip seal samples prepared with mix gradation will be really valuable to verify the SVR approach.

## Data Availability

The data that support the findings of this study are available from the corresponding author upon reasonable request.
